# TBE Vaccination Breakthrough Cases—Does Age Matter?

**DOI:** 10.3390/vaccines9080932

**Published:** 2021-08-21

**Authors:** Heinz-J. Schmitt, Gerhard Dobler, Dace Zavadska, Zane Freimane, Dimitrios Fousteris, Wilhelm Erber, Luis Jodar, Andreas Palmborg

**Affiliations:** 1 Medical Development, Scientific and Clinical Affairs, Pfizer Vaccines, Collegeville, PA 19426, USA; qi.yan2@pfizer.com (H.-J.S.); Luis.Jodar@pfizer.com (L.J.); 2 Department of Virology and Rickettsiology, German National TBE Consiliary Laboratory, Bundeswehr Institute of Microbiology, 85748 Munich, Germany; gerharddobler@msn.com; 3 Department of Paediatrics, Children’s Clinical University Hospital, Riga Stradiņš University, LV-1007 Riga, Latvia; dace.zavadska@rsu.lv (D.Z.); Zane.Freimane@rsu.lv (Z.F.); 4 Global Medical, Scientific and Clinical Affairs, Pfizer Vaccines, 1210 Vienna, Austria; fousteris.dimitrio@gmail.com (D.F.); Wilhelm.Erber@pfizer.com (W.E.); 5 Medical Development and Scientific Affairs, Pfizer Vaccines, 19138 Stockholm, Sweden

**Keywords:** tick-borne encephalitis, vaccine failure, vaccination failure, older adults, Sweden

## Abstract

Tick-borne encephalitis (TBE) vaccines are highly effective in preventing TBE and vaccine failures (VF) are rare events. In this study, we compared the age distribution of TBE cases and TBE VF in three endemic countries: Sweden, Southern Germany, and Latvia. While the age distribution of TBE cases was similar for those <50 years versus those ≥50 years in all three countries, in Sweden, a higher proportion of VF cases was ≥50 years, whereas most VF cases in Latvia were <50 years of age and more evenly distributed between those <50 years versus those ≥50 in Southern Germany. Here, theoretical explanations were provided, including differences in diagnostic practices, vaccine uptake between age groups, behavioral patterns and underlying medical conditions, as to why VF were generally older in Sweden than the other countries. There is no scientific rationale to give an extra priming dose of TBE vaccine to subjects ≥50 years of age.

## 1. Introduction

Tick-borne encephalitis (TBE) is an infectious disease of the central nervous system, caused by the TBE virus (TBEV), a flavivirus, which is transmitted by infected ticks or rarely by ingestion of unpasteurized dairy products. As recently reviewed in detail by Dobler et al., three main TBEV subtypes (European, Siberian, and Far-Eastern) and two proposed subtypes (Baikalian, Himalayan) are found in the endemic areas reaching from the United Kingdom, France and Norway in the West to northern Italy in the South, and throughout Central and Eastern Europe to Asia with Japan in the East [1]. Persisting neurological and/or psychiatric sequelae of TBE can be detected in up to 50% of patients [1]. The case fatality rate varies by region and ranges between 0.4 and 6% in central Europe and is up to 20% in Russia [1]. While there is no specific treatment, TBE is preventable by vaccination. There are six licensed vaccines globally, two of them (FSME-IMMUN, Pfizer; Encepur, Bavarian Nordic, formerly GSK) are licensed and available in the European Union. A recent manuscript from Stockholm County reported that among 1004 TBE cases diagnosed between 2006 and 2015, a total of 53 had been fully vaccinated against the disease [2]. As 43 cases (81%) of the vaccine failures (VF) occurred in patients aged >50 years with a median age of 62 years (underlying diseases: 51%), the authors recommend giving an additional priming dose of TBE vaccine to all persons ≥50 years [2]. Here, the age distribution of TBE VF from other data sources was reviewed.

## 2. Materials and Methods

VF was defined as a patient with “reported TBE” despite having received ≥2 doses of either FSME-IMMUN or Encepur at least 2 weeks prior to the onset of disease symptoms and with the last dose given according to the vaccine labels. A systematic review of the literature on TBE VF (Freimane et al., manuscript in preparation) revealed 18 articles; 15 of them were case reports or smaller single-center-based case series, included largely overlapping patient cohorts, or did not contain any detailed age distribution data and were thus not further considered. The three remaining studies which included the comparison of the age distribution of TBE VF were the retrospective study from southern Sweden [2], as well as two long-term population-based cohorts with notified cases, one each from the Bayern and from Baden-Wurttemberg regions of Southern Germany. Data on TBE cases and TBE VF were provided upon request by the respective public health institutes, the National TBE Reference Center in Munich (instmikrobiobw.de/startseite/einrichtungen/konsiliarlabore/konsiliarlabor-fuer-fsme. accessed on 20 November 2020) and the Centre for Disease Prevention and Control of Latvia, Riga, Latvia (www.eurohealthnet.eu/member/centre-disease-prevention-and-control-latvia. accessed on 15 November 2020). Data on TBE cases in Sweden were extracted from the Swedish Public Health Agency database (https://www.folkhalsomyndigheten.se/folkhalsorapportering-statistik/statistik-a-o/sjukdomsstatistik/tick-borne-encephalitis-tbe/?t=county. accessed on 20 November 2020). 

## 3. Results

**Figure 1:**
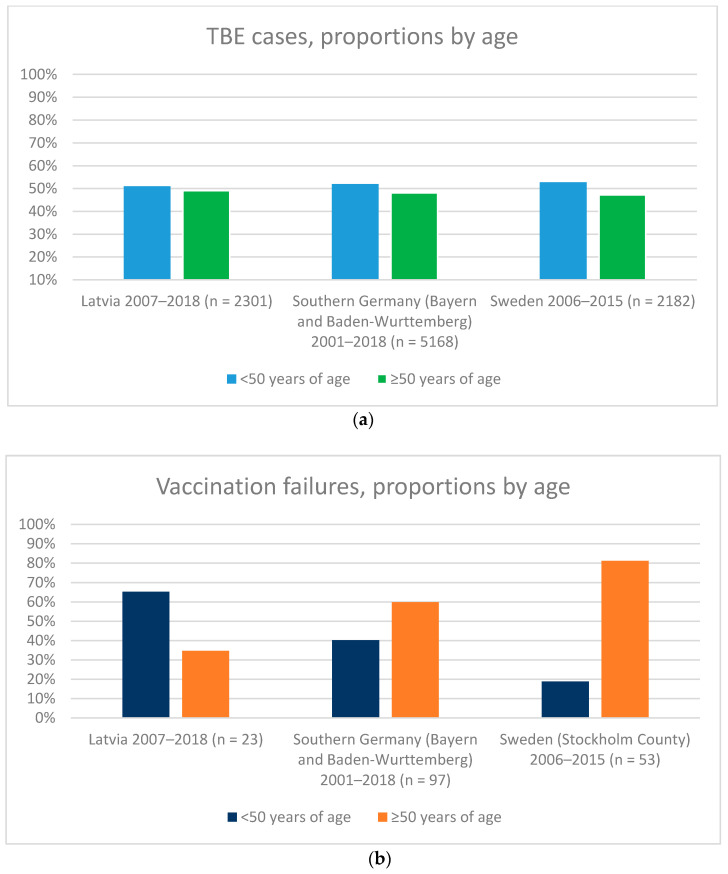
(**a**): Proportion of TBE cases <50 years (blue bars) and ≥50 years (green bars). (**b**): Proportion of TBE VF <50 years (blue bars) and ≥50 years (orange bars). Both panels include data from Latvia, Bayern and Baden-Wurttemberg regions of Germany and Sweden. Data for Latvia were based on Zavadska et al. 2018 and the Centre for Disease Prevention and Control of Latvia, Riga, Latvia (www.eurohealthnet.eu/member/centre-disease-prevention-and-control-latvia. Accessed 15 November 2020). Data for Southern Germany were based on: Dobler et al. 2018; Erber et al. 2021 submitted; and the National TBE Reference Center in Munich (instmikrobiobw.de/startseite/einrichtungen/konsiliarlabore/konsiliarlabor-fuer-fsme. Accessed on 20 November 2020). For Sweden, TBE cases were from the Swedish Public Health Agency (https://www.folkhalsomyndigheten.se/folkhalsorapportering-statistik/statistik-a-o/sjukdomsstatistik/tick-borne-encephalitis-tbe/?t=county. accessed on 20 November 2020). TBE VF in Stockholm were from Hansson et al. 2020.

The analysis of the three data sources identified a total of 173 TBE VF. The median age of TBE VF was 62 years (range: 6–83 years; *n* = 53), 55 years (range: 2–86 years; *n* = 97) and 40 years (range: 1–70 years; *n* = 23) in Southern Sweden, Bayern and Baden-Wurttemberg regions of Germany (southern Germany), and Latvia, respectively. In Latvia out of 23 VFs 15 (65%) occurred in <50 and 8 (35%) in ≥50 years of age, in Southern Germany out of 97 VFs 39 (40%) occurred in <50 and 58 (60%) in ≥50 years of age, whilst in Sweden out of 53 VFs 10 (19%) occurred in <50 and 43 (81%) in ≥50 years of age (Figure 1). In Latvia out of 2301 TBE cases 1174 (51%) occurred in <50 and 1127 (49%) in ≥50 years of age, in Southern Germany out of 5168 TBE cases 2686 (52%) occurred in <50 and 2482 (48%) in ≥50 years of age, and in Sweden out of 2182 TBE cases 1135 (52%) occurred in <50 and 1047 (48%) in ≥50 years of age (Figure 1). Whilst the proportion of TBE cases in <50 years and ≥50 years age groups was similar in the three countries, the proportion of VF differed largely (Figure 1). The proportion of VF was about evenly distributed between <50 years and ≥50 years age groups in Bayern and Baden-Wurttemberg regions of Germany (40% and 60%, respectively), higher in the younger age group in Latvia (65% and 35%, respectively) and disproportionally higher in the older age group in Sweden (19% and 81%, respectively) (Figure 1). 

## 4. Discussion

In this manuscript, we have compared epidemiological features of TBE infections and VF in three European countries (Sweden, Germany and Latvia) and have shown that whereas the TBE infections are evenly distributed between younger and older age groups in the three countries, VF are disproportionally higher in the older age group in Sweden. 

There are several hypotheses that might explain the observed age-specific differences in VF by country. For example, the variability of diagnostic practices in these countries may result in under-diagnosis of TBE in children and young adults versus older adults, possibly due to a milder disease spectrum of the acute disease in younger populations [3,4]. Age-specific variances may also be explained by behavioral patterns. In some countries TBE exposure may differ between younger and older age cohorts, as more senior citizens may live or spend leisure time in the countryside compared to younger ages. Additionally, differences in vaccine uptake between age groups [5,6] or higher proportion of elderly patients with co-morbid diseases or immunosuppressed conditions between countries may also explain these differences. In fact, the label of FSME-IMMUN explicitly states that seroresponse should be checked in immunosuppressed patients after TBE vaccination and that an additional dose should be given if there is no evidence of seroconversion [7]. Finally, disparities may be due to the combination of several or all the factors mentioned above.

Regardless of the underlying conditions that might explain a higher proportion of VF in Sweden, the question remains around the need of administering an additional priming TBE vaccine dose to individuals older than 50 years of age [2]. 

Seroresponse rates induced by FSME-IMMUN after the primary vaccination schedule in subjects 70–87 years of age is 99.3% [8] and there is no evidence that an additional priming dose could be of “immunological” or clinical benefit. Nevertheless, as for other vaccines, antibody titers wane over time [9]. The excellent boostability of priming with two doses of FSME-IMMUN has been documented for all age groups, including subjects ≥60 years, even as long as 20 years after the last vaccine dose [10]. Furthermore, population-based field effectiveness data from Austria indicate a high vaccine effectiveness of >94% in persons ≥61 years [11], and similarly high effectiveness rates were recently confirmed in a separate study based on data from Germany and Latvia (W Erber et al., Effectiveness of TBE vaccinations in Southern Germany and Latvia. Manuscript submitted for publication). High effectiveness and low rates of VFs have also been demonstrated in Switzerland, where even a prolonged booster interval—10 years—is officially recommended, instead of every 5 or 3 years in other countries [7,12].

## 5. Conclusions

In conclusion, TBE VF are very rare events—173 VF cases described in this review—which relates favorably to more than 160 million doses of TBE vaccines globally distributed since 1976. Whilst the higher proportion of VF in older age groups observed in Sweden warrants further investigation, the current TBE primary vaccination schedule per age group as described in the label remains the optimal choice to be continued. 

## Data Availability

The data that support the findings of this study are available from the corresponding author, A.P., upon reasonable request.
